# Analysis and Suppression of Thermal Magnetic Noise of Ferrite in the SERF Co-Magnetometer

**DOI:** 10.3390/ma15196971

**Published:** 2022-10-07

**Authors:** Haoying Pang, Feng Liu, Wengfeng Fan, Jiaqi Wu, Qi Yuan, Zhihong Wu, Wei Quan

**Affiliations:** 1 School of Instrumentation and Optoelectronic Engineering, Beihang University, Beijing 100191, China; 2 Zhejiang Provincial Key Laboratory of Ultra-Weak Magnetic-Field Space and Applied Technology, Hangzhou Innovation Institute, Beihang University, Hangzhou 310051, China; 3 Innovative Research Institute of Frontier Science, Beihang University, Beijing 100191, China

**Keywords:** ferrite complex permeability, low frequency, SERF co-magnetometer, thermal magnetic noise

## Abstract

The ferrite magnetic shield is widely used in ultra-high-sensitivity atomic sensors because of its low noise characteristics. However, its noise level varies with temperature and affects the performance of the spin-exchange relaxation-free (SERF) co-magnetometer. Therefore, it is necessary to analyze and suppress the thermal magnetic noise. In this paper, the thermal magnetic noise model of a ferrite magnetic shield is established, and the thermal magnetic noise of ferrite is calculated more accurately by testing the low-frequency complex permeability at different temperatures. A temperature suppression method based on the improved heat dissipation efficiency of the ferrite magnetic shield is also proposed. The magnetic noise of the ferrite is reduced by 46.7%. The experiment is basically consistent with the theory. The sensitivity of the co-magnetometer is decreased significantly, from 1.21 × $10^{-5}$°/s/Hz${}^{1/2}$ to 7.02 × $10^{-6}$°/s/Hz${}^{1/2}$ at 1 Hz. The experimental results demonstrate the effectiveness of the proposed method. In addition, the study is also helpful for evaluating the thermal magnetic noise of other materials.

## 1. Introduction

Atomic magnetometers and co-magnetometers based on a spin-exchange relaxation-free (SERF) regime have been widely used in frontier science research [1,2], ultra-weak magnetic field measurement [3,4,5], inertial navigation [6,7] and magnetoencephalography [8,9] because of their ultra-high-sensitivity magnetic field and inertial measurement capability. In particular, the SERF co-magnetometer has the potential for high accuracy, miniaturization and low cost, and has been seen as the development direction for the next generation of rotational sensors [10,11,12]. The passive magnetic shield is a crucial part of the co-magnetometer [13]. It is usually made of μ-metal material with high magnetic permeability, which shields the external environment from electromagnetic interference and provides the necessary low magnetic field environment for the SERF co-magnetometer [14,15].

Although passive magnetic shields can provide adequate shielding against external interference, the shielding material generates magnetic noise due to Johnson currents, which becomes a limiting factor in improving the sensitivity of SERF co-magnetometers [16,17]. In recent years, ferrite materials have been used as inner shielding materials for SERF co-magnetometers and magnetometers due to their high resistivity and low loss [18]. Kornack et al. first used ferrite in the SERF magnetometer. The noise level reached 0.75 fT/Hz${}^{1/2}$, 25 times lower than the μ-metal material [19]. Since then, ferrite has been widely used in various high-sensitivity atomic sensors, such as atomic magnetometers [5], nuclear magnetic resonance gyroscopes [20], etc. In 2019, Fan et al. used a ferrite magnetic shield in a SERF co-magnetometer and noted that ferrite with low magnetic noise and high temperature stability is the superior inner shielding material [16].

Many researchers have studied the noise characteristics of ferrite to reduce magnetic noise further [21,22]. The magnetic noise of cylindrical ferrite was calculated using the fluctuation–dissipation theorem created by Kornack [19]. They pointed out that the magnetic noise of ferrite is mainly related to its complex permeability, temperature and size. Subsequently, Lee et al. performed calculations for shields with simple geometries [23]. In addition, Ma et al. developed a more accurate model to analyze the longitudinal and transverse magnetic noise of the ferrite [24]. The magnetic noise is also effectively reduced by optimizing the dimensional parameters of the cylindrical ferrite shield. Yang proposed a more accurate method for measuring the complex permeability of ferrite [25]. In addition, for large-size ferrite, Lu studied the magnetic noise of the multi-annular ferrite shield and pointed out that the effect of the gap is negligible when the gap width is less than 0.01 mm [26]. However, the temperature properties of the material have not been studied in the literature. The characteristics of high-temperature superconducting materials [27] and soft magnetic materials are affected by temperature. Chrobak et al. obtained the temperature dependence of the critical current of the film from the temperature dependence of the imaginary part of the AC susceptibility of the high-temperature superconducting material [28]. Additionally, Fiorillo et al. analyzed the loss and permeability versus temperature in soft magnetic ferrites [29]. This does not apply to the low-frequency range required by the SERF co-magnetometer. Therefore, our work needs to consider the effect of temperature on magnetic noise at low frequencies and the variation in ferrite complex permeability at different temperatures.

In this paper, the thermal magnetic noise of ferrite is analyzed theoretically, and a temperature suppression method based on improving the heat dissipation efficiency is proposed. First, we tested the complex permeability of the ferrite at different temperatures and calculated the variation in magnetic noise according to temperature. After that, a SERF magnetometer was used to compare the magnetic noise before and after the ferrite temperature suppression. The theory’s accuracy was verified by the basic agreement between theory and experiment. Finally, the sensitivity of the co-magnetometer system was tested, and it was experimentally demonstrated that the sensitivity performance of the system could be significantly improved by reducing the ferrite temperature.

## 2. Methods

According to the generalized Nyquist theory, the random motion of the electric charge caused by the thermal motion of atoms in a conductor will produce an arbitrary change in current, and the change in current will produce a magnetic field [23,30]. Therefore, atomic thermal motion inside the ferrite generates magnetic interference in the shielded space, and this interference is the primary source of magnetic noise in the SERF co-magnetometer. The fluctuation–dissipation theorem is used to calculate the magnetic noise. Assuming that an excitation coil with an applied oscillating current is placed in a ferrite shield and another pickup coil is placed in the same position, the total power loss in the shielded cylinder is defined as P(f). The relationship between the magnetic noise δB(f) and P(f) is as follows:

(1) $$\delta B\left(f\right)=\frac{\sqrt{4k_{B}T}\sqrt{2P(f)}}{2\pi fAI},$$

where $k_{B}$ is the Boltzmann constant, *T* is the absolute temperature of the shields, *A* is the area of the excitation coil and *I* and *f* are the current and frequency of the excitation coil. P(f) contains eddy-current loss, hysteresis loss and excess loss. For the ferrite shield, its power loss at low frequency is mainly hysteresis loss, so the expression of P(f) is

(2) $$P\left(f\right)\approx P_{hyst}\left(f\right)=\int_{V}\pi f\mu^{\prime \prime }H^{2}dV,$$

where $P_{hyst}\left(f\right)$ is the hysteresis loss and *V* is the volume of the magnetic shield. $\mu =\mu^{\prime }-i\mu^{\prime \prime }$ is the complex permeability of the magnetic material; $\mu^{\prime }$ and $\mu^{\prime \prime }$ are the real and imaginary parts of the complex magnetic permeability, respectively. *H* is the magnetic field strength. The above equation represents the volume integral of the magnetic shield in an oscillating magnetic field with an amplitude of *H*.

Considering the ferrite magnetic shield as a long and hollow cylinder, the longitudinal magnetic field noise in the shield is [19]

(3) $$\delta B\left(f\right)=\frac{0.26\mu_{0}}{r\sqrt{t}}\sqrt{\frac{2k_{B}T\mu^{\prime \prime }}{\pi f\mu {}^{\prime 2}}},$$

$\mu_{0}$ is the vacuum permeability, and *r* and *t* are the inner diameter and thickness of the shield. The noise in this equation is directly related to the temperature. In addition, the complex permeability of the ferrite increases with increasing temperature. Therefore, the analysis of the thermal magnetic noise of ferrite requires accurate testing of the low-frequency complex permeability at different temperatures.

The coils are wound uniformly around the ferrite sample, corresponding to an equivalent resistive and inductive circuit. The complex permeability of the ferrite can be measured by measuring the inductance and resistance parameters of the circuit. The relationship between complex permeability, inductance and resistance is as follows (see Appendix A for details):

(4) $$L_{s}=\frac{\mu_{0}A_{e}N^{2}}{l_{e}}\mu^{\prime },$$

(5) $$R_{y}=\frac{2\pi f_{c}\mu_{0}A_{e}N^{2}}{l_{e}}\mu^{\prime \prime }+R_{w},$$

where $L_{s}$ is the inductance of the sample, $l_{e}$ is the effective magnetic circuit length, $A_{e}$ is the effective cross-sectional area, *N* is the number of turns of the winding, $R_{y}$ is the resistance value containing the resistance of the test coil, $R_{w}$ is the resistance value of the measurement coil and *f* is the test frequency.

The complex magnetic permeability at a specific magnetic field strength was obtained using the above method. However, the complex permeability at zero magnetic field conditions is required for theoretical calculations. Under small magnetic field excitation, the complex permeability is related to the magnetic field strength as $\mu_{h}=\mu_{a}+\eta H$. Here, $\mu_{h}$ is the amplitude permeability, $\mu_{a}$ is the permeability at zero fields and η is the Rayleigh constant. Therefore, in this paper, the complex permeability is tested at different magnetic field strengths, and then the complex permeability is obtained at zero field conditions based on the above relationship.

## 3. Experimental Setup

The experimental setup in this section is divided into two parts. First, a complex permeability test system is built to test the complex permeability of ferrite at different temperatures and quantitatively analyze the relationship between magnetic noise and temperature. Then, the K-Rb-${}^{21}$Ne co-magnetometer system is built to test the effectiveness of the thermal and magnetic noise suppression method.

**Figure 1:**
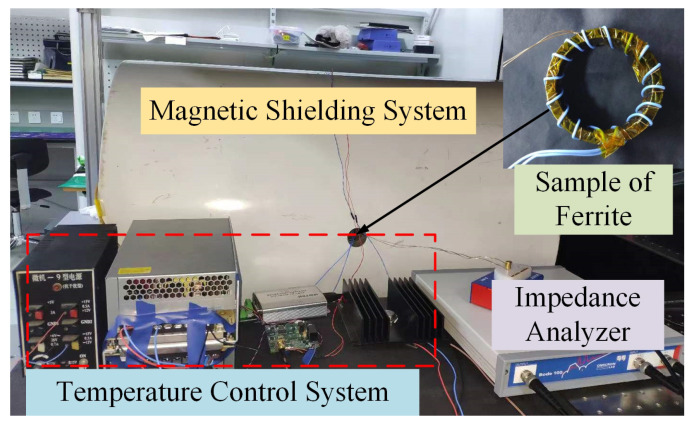
Experimental test system for temperature characteristics of ferrite complex permeability.

The ferrite complex permeability test system is shown in Figure 1. The magnetic shielding system is used to shield the external environment from magnetic field interference and contains an oven, a sample of ferrite wound with demagnetizing and enameled wires. The demagnetizing wire is used to demagnetize the sample, and the enameled wire is used as the test coil. The inner diameter of the ferrite sample is 15 mm, the outer diameter is 25 mm, the height is 7.5 mm and the permeability is 2000. A self-made temperature control system is used to control the temperature of the sample, and the temperature control accuracy is 10 mK. The Omicron-lab BODE 100 impedance analyzer is used to apply different excitation intensities to the sample and to measure the inductance and resistance. The range of magnetic excitation applied to the sample is 0.3–0.8 A/m. To reduce the influence of remanence, the shielding system and the sample are demagnetized, respectively. Furthermore, the temperature is changed and left for at least half an hour to allow the sample to equilibrate with the ambient temperature. The measurements are repeated ten times for each set of conditions to ensure the precision of the test.

**Figure 2:**
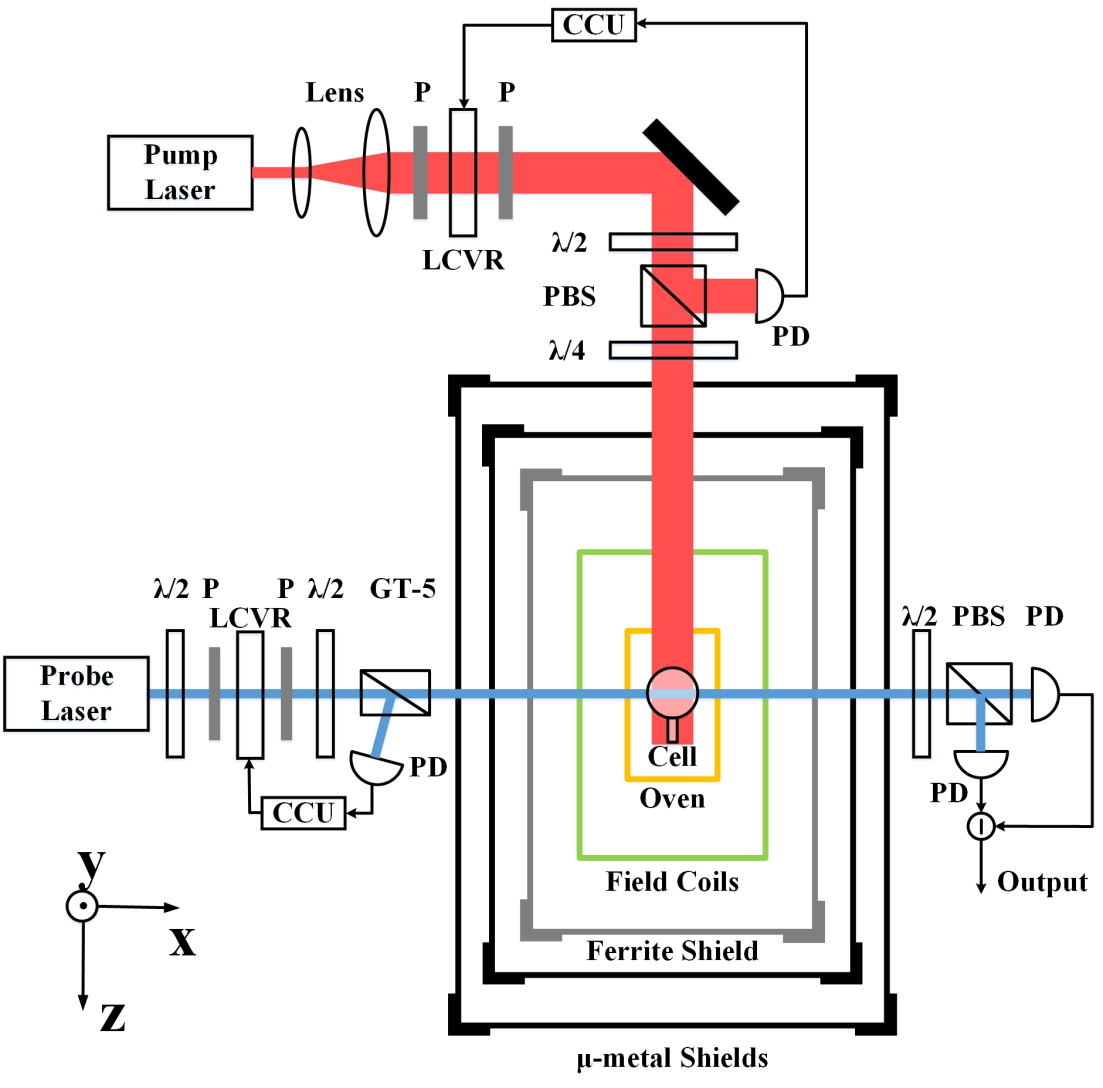
Schematic diagram of the co-magnetometer system. P: polarizer; LCVR: liquid crystal variable retarder; CCU: circuit control system; λ/2: half wave plate; PBS: polarizing beam splitter; λ/4: quarter wave plate; PD: photodiode; GT-5: Glan–Taylor polarizer.

The schematic diagram of the K-Rb-${}^{21}$Ne co-magnetometer system is shown in Figure 2. The magnetic shielding system consists of the outer layer of the Permalloy shield and the inner layer of the ferrite shield. Their thicknesses are 2 mm and 6 mm, respectively. The outer shield has a permeability of about 20,000. It is used to shield the external ambient magnetic field, but its large eddy current loss results in a relatively high level of magnetic noise in the system. The inner ferrite shield is used to shield the magnetic noise of Permalloy. Therefore, the magnetic noise of the system is mainly the magnetic noise of the ferrite. This is the prominent noise studied in this paper. Inside the magnetic shielding system are coils, an oven, and an alkali metal vapor cell. Coils are used to compensate for the residual magnetic field inside the shield. The oven heats an 8 mm diameter GE180 aluminosilicate vapor cell to an operating temperature of 180 °C. External to the magnetic shielding system is the optical system, which provides the pump and probe lights necessary for the operation of the co-magnetometer. When the system is operated in SERF magnetometer mode, the vapor cell contains a mixture of K and Rb alkali metals, 50 Torr of N${}_{2}$ quenching gas and 2 atm of ${}^{4}$He buffer gas. The SERF magnetometer is used to test the magnetic noise level of the system. When the co-magnetometer system operates in gyroscope mode, only the vapor cell needs to be replaced. At this time, the vapor cell contains a mixture of K and Rb alkali metals, 2 atm of ${}^{21}$Ne and 50 Torr of N${}_{2}$. The co-magnetometer system is used to verify the effectiveness of the thermal magnetic noise analysis and suppression method proposed in this paper.

**Figure 3:**
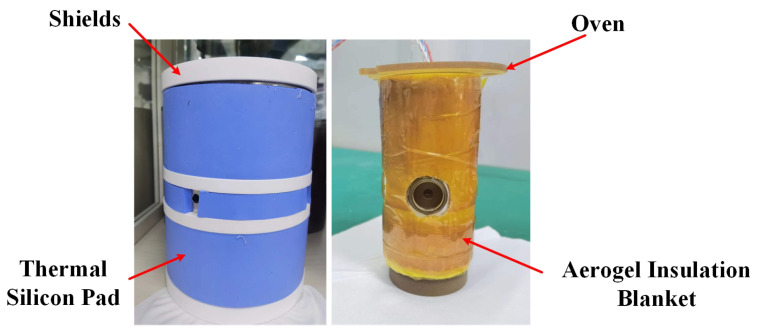
Temperature suppression method for the magnetic shield.

The temperature near the vapor cell of the co-magnetometer system is as high as 180 °C, while the whole system is at an ambient temperature of 25 °C. The temperature is reduced from high to low temperature by heat conduction and heat convection. The ferrite shield is part of the heat dissipation path, and its temperature is higher than the ambient temperature. The closer it is to the vapor cell, the higher its temperature. Because the performance of the co-magnetometer system is related to the volume of the magnetic shield and the vapor cell temperature, the temperature of the ferrite shield cannot be reduced by changing the cell temperature and the volume of the magnetic shield. Therefore, this paper uses the change in heat dissipation efficiency to reduce the temperature of the ferrite and suppress the thermal magnetic coupling noise. The exact method is shown in Figure 3. On the one hand, a high thermal conductivity silicon pad is wrapped around the outside of the magnetic shield to improve the heat dissipation efficiency of the shield. The thermal silicone pad has a thermal conductivity of 4 W/(m·K). On the other hand, the aerogel insulation blanket is wrapped around the outside of the oven, and the thermal conductivity of the aerogel insulation blanket is 0.02 W/(m·K), which reduces the heat transfer from the cell to the shield. This method utilizes the gaps between the magnetic shields and does not negatively affect the original system. Solutions filled with thermally conductive or insulating materials have a wide range of engineering applications, such as nuclear magnetic resonance gyroscopes.

## 4. Results and Analysis

### 4.1. Measurement of Ferrite Complex Permeability at Different Temperatures

**Figure 4:**
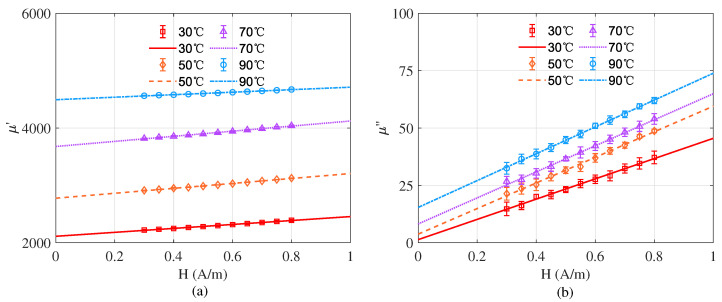
(**a**) Real part of permeability at different temperatures. (**b**) Imaginary part of permeability at different temperatures.

First, the BODE 100 impedance analyzer is used to apply a current *I* = 1.13 mA to the test coil, producing the corresponding magnetic field strength *H* = 0.3 A/m. BODE 100 is also used to measure resistance $R_{y}$ and inductance $L_{e}$ in the frequency range of 20–200 Hz. According to Equation (4), the real part of the magnetic permeability can be calculated. The imaginary part of the permeability $\mu {}^{\prime \prime }$ and the resistance of the wire $R_{w}$ can be obtained by linear fitting the test data according to Equation (5). After that, to get the complex permeability under zero magnetic field excitation, the complex permeability under different excitations are measured. The range of current *I* applied to the test coil is 1.13 mA to 3.76 mA, and the corresponding magnetic field strength is 0.3 to 1 A/m. By linear fitting the magnetic field strength *H* and the permeability, the permeability at zero magnetic field strength can be obtained. The complex permeability is tested at 30 °C, 50 °C, 70 °C and 90 °C for different intensity excitations, respectively. As shown in Figure 4, the different marks indicate the actual measured values of complex permeability at different temperatures, the lines indicate the fitted curves of the actual measured values and the error bars are the standard deviations of 10 times test at different temperatures. The complex permeability at zero excitation field at different temperatures is obtained from the fitted curves and is summarized in Table 1. Equation (3) is used to calculate the magnetic noise according to the size of the ferrite in the co-magnetometer. The inner and outer diameters of the ferrite magnetic shield are 72 mm and 84 mm, respectively. The theoretical magnetic noise at 1 Hz is shown in Table 1. When the ferrite temperature is reduced from 90 °C to 30 °C, the magnetic noise at 1 Hz is reduced from 12.93 fT/Hz${}^{1/2}$ to 7.45 fT/Hz${}^{1/2}$.

### 4.2. Magnetic Noise Measurement

In this paper, we reduce the temperature of the magnetic shield by changing the heat transfer in the co-magnetometer, increasing the insulation of the oven as well as improving the efficiency of heat dissipation. When the magnetic shield temperature is not suppressed, the shield temperature is 79.1 °C, and the shield temperature after suppression is 33.2 °C.

**Figure 5:**
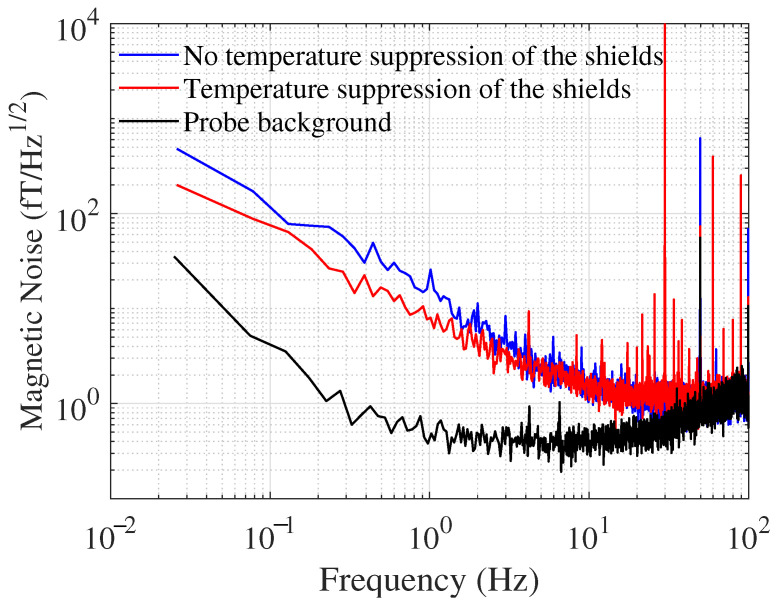
Magnetic noise measurement results of SERF magnetometer.

Due to the extremely low conductivity of ferrite, its eddy current loss below KHz can be disregarded, and the hysteresis loss magnetic noise becomes the dominant low-frequency magnetic noise in the SERF magnetometer, which can be regarded as 1/f noise. In this subsection, the magnetic noise of the system is tested using a SERF magnetometer. The magnetic noise is tested before and after the temperature suppression of the shield, respectively. Figure 5 indicates the magnetic noise curves of the magnetometer in both cases. For comparison, the probe background noise of the system (solid black line) is also tested. The probe background noise is significantly lower than the sensitivity curve of the magnetometer, indicating that the sensitivity of the magnetometer is primarily limited by the magnetic noise. Meanwhile, in the low-frequency band, the system sensitivity curve shows a significant 1/f noise, indicating that it is primarily due to the hysteresis loss magnetic noise of the ferrite. The solid blue line and the solid red line show the sensitivity curves before and after temperature suppression of the shield, respectively. The system sensitivity is 15.24 and 8.12 fT/Hz${}^{1/2}$ at 1 Hz, respectively. The noise at 30 Hz is due to the application of a calibrated magnetic field with a peak-to-peak value of 0.5 nT, and the noise at 50 Hz is the power frequency noise of the circuit. As the temperature increases, the intense thermal motion of the atoms inside the sample prevents the magnetic moment of the atoms from varying with the applied magnetic field, leading to an increase in the Rayleigh constant. Thus, the hysteresis loss increases, leading to an increase in the magnetic noise of the system. The magnetic noise of the system is reduced by 46.7% by the shield temperature suppression method proposed in this paper. The experiments basically agree with the theory, and the magnetometer test results are slightly larger than the calculated results in the previous subsection. This difference may be due to the openings in the ferrite and the gap between the ferrite annulus.

### 4.3. Sensitivity Measurement of SERF Co-Magnetometer

Finally, to demonstrate the accuracy of the theoretical analysis and the effectiveness of the suppression method, the sensitivity is tested in a SERF co-magnetometer.

**Figure 6:**
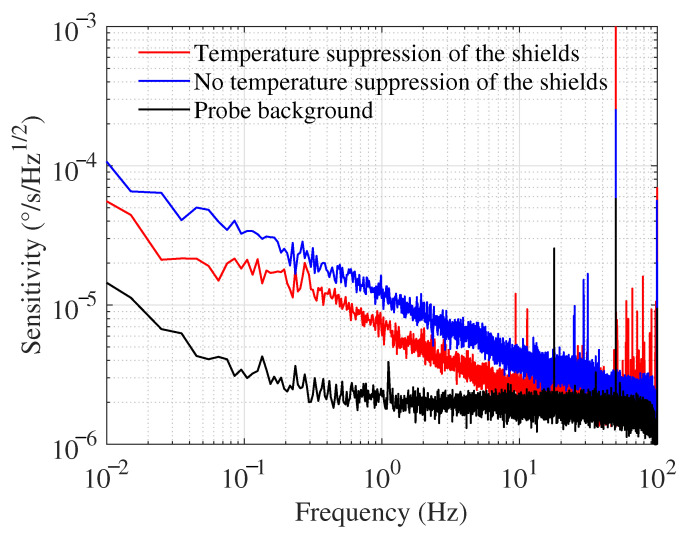
SERF magnetometer sensitivity measurement results.

The system signal error caused by magnetic noise can be expressed as

(6) $$N_{B}\left(f\right)=\delta B\left(f\right)\left|G_{B}\left(f\right)\right|,$$

where $\left|G_{B}\left(f\right)\right|$ is the amplitude–frequency response of the system to the magnetic field [31]. When the magnetic noise of a co-magnetometer system is reduced, its inertial measurement sensitivity is reduced accordingly. The probe background noise of the system is tested, as shown by the solid black line in Figure 6, with a noise value of 2.21 × $10^{-6}$°/s/Hz${}^{1/2}$ at 1 Hz. The probe background noise is much lower than the system noise, indicating that the system is in a normal inertial measurement state, so the system noise reduction provided by the ferrite magnetic noise reduction will not be annihilated by the probe background noise. The blue and red solid lines in Figure 6 indicate the sensitivity curves of the co-magnetometer system before and after temperature suppression of the ferrite shield, respectively. Since the bandwidth of the SERF co-magnetometer is within 5 Hz, this work focuses on the sensitivity at low frequencies. After the ferrite temperature suppression, the system’s noise is significantly reduced in the 5 Hz range. The system’s sensitivity is reduced from 1.21 × $10^{-5}$°/s/Hz${}^{1/2}$ to 7.02 ×$10^{-6}$°/s/Hz${}^{1/2}$ at 1 Hz. The sensitivity performance of the system is improved by 42.0%.

## 5. Conclusions

An accurate method for analyzing and measuring the thermal magnetic noise of ferrite in the SERF co-magnetometer is proposed for the first time, and a suppression method is proposed accordingly, which significantly improves the measurement sensitivity of the co-magnetometer. Firstly, a thermal magnetic noise model of ferrite is established, and a low-frequency complex permeability test method at different temperatures is proposed. The complex permeability test system and the co-magnetometer system are built to test the thermal magnetic noise of ferrite, respectively. The results show that the measured and theoretical results of magnetic noise at different temperatures are basically consistent. The magnetic noise of the ferrite at 1 Hz is reduced from 15.24 fT/Hz${}^{1/2}$ to 8.12 fT/Hz${}^{1/2}$ by the proposed temperature suppression method. This study can not only evaluate the thermal magnetic noise of other low-noise magnetic materials, but is also essential to improve the sensitivity of SERF co-magnetometer and magnetometer. 

## Figures and Tables

**Table 1:** Variation in ferrite complex permeability with temperature.

Temperature (${}^{{}^{\circ}}$C)	$\mu {}^{\prime }$	$\mu {}^{\prime \prime }$	δB(f) at 1 Hz (fT/Hz${}^{1/2}$)
30	2113.4	1.37	7.45
50	2777.3	3.77	9.77
70	3679.0	8.23	11.22
90	4494.6	15.42	12.93

## Data Availability

Not applicable.

## Sample Availability

The data presented in this study are available on request from the corresponding author.

## Abbreviations

SERF	Spin-Exchange Relaxation Free

## Appendix A

### Principle of Complex Magnetic Permeability Measurement

According to the measurement principle of the coil method, the relationship between the complex permeability of the ferrite and the inductance and resistance in the equivalent circuit is as follows:

(A1) $$\mu^{\prime }=\frac{l_{e}L_{s}}{\mu_{0}A_{e}N^{2}},$$

(A2) $$\mu^{\prime \prime }=\frac{l_{e}\left(R_{y}-R_{w}\right)}{2\pi f_{c}\mu_{0}A_{e}N^{2}}.$$

The definition of each variable in the formula is shown in the following table:

**Table A1:** Definition of variables.

Trem	Description
$L_{s}$	inductance of the sample
$l_{e}$	effective magnetic circuit length
$A_{e}$	effective cross-sectional area
*N*	number of turns of the winding
$R_{y}$	resistance value containing the resistance of the test coil
$R_{w}$	resistance value of the measurement coil
*f*	test frequency

The expressions of $l_{e}$ and $A_{e}$ are:

(A3) $$\begin{array}{c} l_{e}=\frac{\pi \ln(D_{y}/d_{y})}{{d_{y}}^{-1}-{D_{y}}^{-1}},\;\\ A_{e}=\frac{h_{y}\ln^{2}\left(D_{y}/d_{y}\right)}{2\left({d_{y}}^{-1}-{D_{y}}^{-1}\right)}, \end{array}$$

where $D_{y}$ is the outer diameter of the sample, $d_{y}$ is the inner diameter, and $h_{y}$ is the height of the sample.

The real part of the magnetic permeability can be calculated directly from the inductance value. The imaginary part contains the resistance value of the measurement coil, and Equation (A2) needs to be rewritten as

(A4) $$R_{y}=\frac{2\pi f_{c}\mu_{0}A_{e}N^{2}}{l_{e}}\mu^{\prime \prime }+R_{w}.$$

By fitting the above equation to $f_{c}$ and $R_{y}$, the slope and intercept are the imaginary part of the magnetic permeability and the resistance of the measurement coil, respectively.
